# Cross-cultural validation of the integrated palliative outcome scale for neurological patients (IPOS-Neuro S8) in multiple sclerosis patients

**DOI:** 10.1017/S1478951525000392

**Published:** 2025-06-03

**Authors:** Kim Dillen, Wiebke Müller, Martin Hellmich, Yasemin Goereci, Veronika Dunkl, Anne Dorr, Gereon R. Fink, Raymond Voltz, Mevhibe Hocaoglu, Clemens Warnke, Heidrun Golla

**Affiliations:** 1 Department of Palliative Medicine, University of Cologne, Faculty of Medicine and University Hospital, Cologne, Germany; 2 Institute of Medical Statistics and Computational Biology (IMSB), University of Cologne, Faculty of Medicine and University Hospital, Cologne, Germany; 3 Department of Medical Statistics, University Medical Center Göttingen, Göttingen, Germany; 4 Department of Neurology, University of Cologne, Faculty of Medicine and University Hospital, Cologne, Germany; 5 Cognitive Neuroscience, Institute of Neuroscience and Medicine (INM-3), Research Centre Jülich, Jülich, Germany; 6 Faculty of Medicine and University Hospital, Center for Integrated Oncology Aachen Bonn Cologne Düsseldorf (CIO ABCD), University of Cologne, Cologne, Germany; 7 Faculty of Medicine and University Hospital, Center for Health Services Research (ZVFK), University of Cologne, Cologne, Germany; 8 Policy & Rehabilitation, Cicely Saunders Institute of Palliative Care, King’s College London, London, UK; 9 Philipps University Marburg and Department of Neurology, University Hospital Gießen and Marburg, Baldingerstraße, Marburg, Germany; 10 Department of Palliative Medicine, University Medical Center Göttingen, Göttingen, Germany

**Keywords:** German, Palliative care concerns, symptom burden, prospective observational design, outcome measurement

## Abstract

**Objectives:**

Standardized measures to evaluate neurological patients in palliative care are missing. The Integrated Palliative Outcome Scale, a self-report tailored for neurological patients (IPOS Neuro-S8) helps identify symptom burden but lacks validation in German. This study aimed to validate the IPOS Neuro-S8 in severely affected multiple sclerosis (MS) patients.

**Methods:**

This validation study is a secondary analysis of data from a clinical phase II intervention study with severely affected MS patients. The original study enrolled German-speaking patients aged 18 with severe MS who receive an escalating immunotherapeutic agent and/or exhibit a high level of disability were recruited from the administrative district Cologne (#DRKS00021783). In this validation study, we evaluated construct, discriminant, and convergent validity, internal consistency, test–retest reliability, and sensitivity to change of the IPOS Neuro-S8, using the “Hamburger Lebensqualitätsmessinstrument” (HALEMS), and the Hospice and Palliative Care Evaluation supplemented by neurological symptoms (HOPE+) as comparison measures.

**Results:**

Data from 80 MS patients (mean age 56, SD = 11) were analyzed. Exploratory and confirmatory factor analyses revealed a 3-factor structure (*r* = 0.34–0.63), reflecting distinct clinical patterns, i.e., *breath-mouth connection, pain-sleep cycle*, and *nausea-vomiting link*. Significant convergent validity to hypothesized total score of the HOPE+ (*r_s_*(78) = 0.71, *p* < 0.001) and good discriminant validity using the HALEMS total score (*r_s_*(78) = 0.48, *p* < 0.001) were observed. Correlation with physical symptoms of the HALEMS was stronger than with nonphysical aspects. Internal consistency (Cronbach’s α = 0.67) and test–retest reliability (intraclass coefficient = 0.75) were acceptable.

**Significance of results:**

IPOS Neuro-S8 displays promising psychometric properties for assessing palliative care symptoms in severe MS, a model for other severe neurological diseases due to MS's broad central nervous involvement, allowing findings to be transferable to other neurological diseases. A criterion for minimal clinically important difference was established to evaluate the sensitivity to change. Additional validation across different neurological conditions and disease severities is warranted to enhance generalizability and clinical utility.

## Introduction

Patient-reported outcome measures (PROMs) are invaluable instruments in capturing a patient’s holistic perception of health, encompassing psychological, social, and spiritual dimensions, which is essential for palliative care (Dawson et al. 2010). Widely used to evaluate functional status, quality of life (QoL), and symptomatology, PROMs help focus clinical attention on patients’ primary concerns (Dawson et al. 2010; Bausewein et al. 2016, 2011).

In chronic neurological conditions, PROMS not only assess and track patients’ status but also facilitate the implementation of tailored interventions. Long-term neurological conditions pose complex physical, psychosocial, and spiritual challenges that may necessitate palliative care (Chahine et al. 2008). Due to the widespread symptoms unique to neurological patients, comprehensive assessment tools are recommended for ongoing monitoring throughout their disease trajectory, ensuring a thorough understanding of their evolving health (Wilson et al. 2019; Ciani et al. 2023).

To the best of our knowledge, only two palliative care outcome measurements have been validated to assess burdensome symptoms in patients with long-term neurological conditions: the Hospice and Palliative Care Evaluation supplemented by neurological symptoms (HOPE +) (Dillen et al. 2019) and the Integrated Palliative care Outcome Scale (IPOS) Neuro (Gao et al. 2016; Wilson et al. 2019). The HOPE+ assesses symptom burden *incidence and intensity*, while the IPOS Neuro evaluates the *impact* of symptoms on patients’ daily lives. Recently, the HOPE+ has been validated for use in German-speaking healthcare settings (Dillen et al. 2019). As the only available German-language questionnaire addressing palliative care in neurological patients, HOPE+ serves as the leading gold standard on this subject in Germany. In contrast, the IPOS Neuro, internationally recognized, holds potential for use in global comparisons pending validation in the German healthcare context. The IPOS Neuro is a globally acknowledged, reliable, and valid psychometric instrument developed specifically to monitor outcomes in individuals with progressive long-term neurological conditions. It identifies palliative care concerns in neurological patients at an early stage and aids in establishing appropriate care structures, if indicated. The IPOS Neuro, thus far only available as a self-report version, offers two short adaptations of the full 45-item version, containing 8 and 24 symptom-specific items, demonstrating good psychometric properties (Gao et al. 2016; Wilson et al. 2019). The IPOS Neuro-S8 evaluates symptom burden associated with 8 core symptoms of the full version over the past 3 days, including pain, nausea, vomiting, mouth problems, sleeping difficulties, breathlessness, spasms, and constipation, considering symptom severity and patient perception of impact. This concise tool is suitable for various clinical settings and research contexts. Clinically, it supports early identification of palliative care needs, enabling timely interventions to address symptom burden and improve quality of life for patients with progressive neurological conditions. Its concise design and focus on 8 core symptoms make it suitable for use across diverse care settings, including outpatient, inpatient, and specialized palliative care facilities, while minimizing the burden and time constraints on patients with severe or terminal illnesses. In research, the IPOS Neuro-S8 provides a reliable and standardized method for evaluating symptom outcomes, facilitating longitudinal and comparative studies, and contributing to the development of evidence-based care models.

While validated for English speakers, the tool requires cross-cultural adaptation and validation for non-English-speaking populations. Recently, the authors have culturally adapted the IPOS Neuro-S8 for the German healthcare context (Dillen et al. 2023) (which can be found as Supplemental File S1). In this cultural adaptation study, cognitive interviewing was employed to verify the clarity and accuracy of the instructions and items and to assess face and content validity. This process followed the initial 6 phases outlined in “The Palliative care Outcome Scale (POS) Family of Measures Manual for Translation, Cross-Cultural Adaptation, and Psychometric Testing” (Antunes et al. 2012). In the first phase, the underlying concepts of each item were clarified to ensure alignment with the care concepts of the German culture. The IPOS Neuro-S8 was then translated into German by 2 independent translators with complementary expertise, followed by a back-translation into English by a native English speaker unfamiliar with the original version and without clinical or medical experience. Next, an expert review was conducted to establish conceptual, semantic, experiential, and content equivalence. Subsequently, cognitive interviews with clinical staff and patients assessed the measure’s comprehension, acceptability, clarity, relevance, and length. After completing these steps, all required documents were submitted to the POS Development Team for final review and approval. Overall, all 8 items achieved consensus, although some adjustments were required for certain terms to ensure cultural and conceptual equivalence, particularly for *spasms* and *mouth problems*. Patients and staff found the measure clear, concise, and clinically relevant, with feedback emphasizing the importance of cognitive interviewing in translation processes. Although some respondents questioned the inclusion of specific symptoms and the 3-day recall period, the measure’s core structure was retained to align with the original measure (Dillen et al. 2023). Next, its validity and reliability must be assessed before a standard use in Germany. This procedure will also support multicenter research projects with international partners for comparative analysis.

This study aims to investigate the reliability and validity of the IPOS Neuro-S8 in severely affected MS patients, a representative group of patients with severe neurological diseases which can affect any cell in the central nervous system.

## Methods

### Study design

This validation study is a secondary analysis of all collected data from a clinical phase II intervention study with severely affected multiple sclerosis (MS) patients, following the “The Palliative care Outcome Scale (POS) Family of Measures Manual for Translation, Cross-Cultural Adaptation and Psychometric Testing” (Antunes et al. 2012).

### Setting and participants

The original trial (Communication, Coordination and Security for People with Multiple Sclerosis ) (Golla et al. 2022) was conducted at the Departments of Palliative Medicine and Neurology of the University Hospital Cologne. Patients with severe MS, including those with highly active MS and individuals with primary or secondary chronic progressive MS, who undergo treatment with an escalating immunotherapeutic agent and/or exhibit a high level of disability were recruited from the University Hospital of Cologne, partly preselected by collaborating partner organizations including neurologists, the MS registry of the German MS Society and their location group. A sample size calculation for the parent trial was conducted at *n* = 80 (Golla et al. 2022), which aligns with the POS manual’s (Antunes et al. 2012) recommendation of at least 10 subjects per item of the meausure for a psychometric validation study. All participants had to be 18 years or older and proficient in German, with eligibility confirmed by a trial physician during the informed consent process. Recruitment began in January 2020 and lasted for 12 months.

The trial was approved by the University Hospital of Cologne’s ethics review board (#20-1086, May 28, 2020) and registered in the German Clinical Trials Register (#DRKS00021783, June 30, 2020). It was conducted following the Declaration of Helsinki (World Medical Association 2014). All study participants provided written informed consent. If a patient could not consent, a legal representative proficient in German acted on their behalf.

### Outcome measures

The IPOS Neuro was initially designed for individuals with progressive, long-term neurological conditions and has been condensed into two abbreviated versions, one of them being the IPOS Neuro-S8 (Gao et al. 2016). The IPOS Neuro-S8 consists of 3 key questions, with the second question presenting core symptoms from the full 45-item version, addressing 8 physical symptoms experienced over the past 3 days, including pain, nausea, vomiting, mouth problems, difficulty in sleeping, breathlessness, spasms, and constipation. Responses are categorized from 0 (not at all) to 4 (overwhelmingly), with total scores ranging from 0 to 32. The English version of the IPOS Neuro-S8 has been validated using data from severely affected patients with neurological conditions such as MS, idiopathic Parkinson’s disease, multiple system atrophy, and progressive supranuclear palsy (Gao et al. 2016).

The HOPE+ is a palliative care outcome measurement with an extended view of neurological symptoms, facilitating the identification of palliative concerns among neurological patients (Dillen et al. 2019). It combines the HOPE Symptoms and Problems Checklist and the HOPE-Neuro, covering 6 domains: neuropsychiatric symptoms, intracranial pressure symptomatology, increasing need for care and assistance, psychological burden and strongly associated symptoms, additional physical symptoms, and powerlessness. Response categories in the HOPE+ range from 0 (none) to 3 (severe). As the HOPE+ is the only established palliative care outcome measure specifically designed for neurological patients in Germany, it can be considered the gold standard in this field in Germany. Therefore, we have used it as comparison measure in this study to evaluate convergent validity.

The “Hamburger Lebensqualitätsmessinstrument” (HALEMS), a German adaptation of the Quality of Life Questionnaire for Multiple Sclerosis (HAQUAMS), serves as a disease-specific measure of health-related QoL (Gold et al. 2001). It comprises 44 items, with 28 contributing to subscores reflecting 5 essential aspects of health-related QoL in MS: fatigue/thinking, mobility (lower limb and upper limb), social function, and mood. Scores on this scale range from 1 to 5 (or 7), with higher scores indicating lower QoL. In this study, we primarily utilized the HALEMS to assess and further confirm convergent and discriminant validity.

All measures were administered electronically to the patients as interview-based self-reports at their home during baseline and 3-month follow-up, except for the HOPE+, which was only given at baseline. Data collection was conducted by highly trained and experienced personnel well-versed in the use of all measures.

### Statistical analysis

All statistical analyses were conducted following the COSMIN guidelines (Gagnier et al. 2021) and using two-sided tests, with a significance level set at <0.05. Calculations were done with SPSS Statistics version 28.0.1.1 (IBM Corp 2021). Confirmatory factor analysis (CFA) was performed using Stata version 18.0 (Stata Corp 2023).

Descriptive statistics for total and item-specific scores included means and standard deviations (SDs). Item-level analyses involved frequency distributions presented as percentages. Floor and ceiling effects were assessed by examining the proportion of respondents scoring either the minimum (0) or maximum (4) on each item. An effect was considered present if more than 15% of responses fell at either extreme (Gao et al. 2016).

To evaluate construct validity and explore the underlying dimensions of the IPOS Neuro-S8, an exploratory factor analysis (EFA) was carried out, followed by CFA, as per the methodology described by Gao et al. (2016). The EFA used the maximum likelihood method with varimax rotation, suitable for samples of 5–10 subjects per item (Nguyen and Waller 2022). The optimal number of factors was determined based on eigenvalues >1, in conjunction with examination of the scree plot. Data suitability for factor analysis was assessed with the Kaiser-Meyer-Olkin Measure of Sampling Adequacy (KMO) and Bartlett’s test of sphericity. Factor loading coefficients ≥ 0.3 in magnitude were considered significant. As direct comparison and to further validate our factor extraction, we also employed Velicer’s minimum average partial test and parallel analyses, and re-ran the analysis using the Diagonally Weighted Least Squares (DWLS) technique. The factorial structure from EFA was confirmed through CFA, utilizing fit indices such as chi-squared statistics, root-mean-squared error of approximation (RMSEA) with 95% CI 0.00 (0.00; 0.09), standardized root mean square residual (SRMR), and comparative fit index (CFI) to evaluate model fit. A RMSEA ≤ 0.08 (preferably ≤ 0.06), SRMR ≤ 0.11, and CFI ≥ 0.95 indicate a good model fit (Hu and Bentler 1998; Fan et al. 1999).

Convergent validity was examined by calculating Spearman’s rank correlation coefficient between the IPOS Neuro-S8 and HOPE+ sum scores. The HOPE+ is considered the current gold standard outcome measure for evaluating the palliative symptom burden of neurological patients in Germany, so we expected a strong correlation of *r* ≥ 0.7 to the IPOS Neuro-S8. Correlations less than 0.4 are considered weak, between 0.4 and 0.6 are considered moderate, and greater than or equal to 0.7 as strong (Akoglu 2018).

Convergent and discriminant validity were further evaluated using Spearman’s rank correlation coefficient, with an expectation of a stronger correlation of *r* ≥ 0.7 between the IPOS Neuro-S8 and the physical domain (mobility: upper and lower limb) compared to the nonphysical domain (fatigue/thinking, social function, mood) of the HALEMS with an anticipated low to moderate correlation of *r* < 0.7. The HALEMS total score, which includes domains beyond physical symptoms, was expected to show moderate correlation of *r* = 0.4–0.6 with the IPOS Neuro-S8 total score, which focuses on physical symptoms.

Since McDonald’s Omega (McDonald 1999) could not be calculated for our data due to item covariances that are negative or zero, we considered Cronbach’s alpha (Cronbach 1988) an alternative measure for internal consistency (Orçan 2023). An alpha value of 0.7 or higher was considered acceptable.

Test–retest reliability was explored using the intraclass correlation coefficient (ICC) with baseline and 3-month follow-up data. To eliminate time trends, the data were mean-centered. ICC values below 0.5 indicate poor reliability, 0.5 and 0.75 suggest moderate reliability, 0.75 and 0.9 indicate good reliability, and above 0.9 suggest excellent reliability.

Sensitivity to change was evaluated through regression analysis utilizing the minimal clinically important difference (MCID) of the HALEMS, derived as 0.38 (Golla et al. 2022). Specifically, the regression equation mapped the MCID of the HALEMS (independent variable) to the IPOS Neuro-S8 (dependent variable).

## Results

### Demographic information

A total of 81 individuals were prescreened between January 2020 and January 2021. One patient withdrew consent. The total sample size for the original trial was calculated at *n* = 80 (Golla et al. 2022), thus recruitment was stopped with the inclusion of the final patient. Demographical characteristics of all participating patients can be found in Table 1. A total of *n* = 75 completed the 3-month follow-up. Drop-out was due to illness (*n* = 4) and death (*n* = 1).

**Table 1.** Demographic data at baseline (*n* = 80)

	*n*	%
Age (years)		
Mean ± Standard deviation	56 ± 11	
Range	26 − 83	
Sex		
Male	28	35
Female	52	65
Diagnosis		
Highly active MS	22	27.5
Primary chronic progressive MS	25	31.3
Secondary chronic progressive MS	33	41.1
IPOS Neuro-S8		
Mean ± Standard deviation	8.0 ± 4.8	
Range	0 − 23	
EDSS		
4.5	2	2.5
5	6	7.5
5.5	3	3.8
6	19	23.8
6.5	17	21.3
7	10	12.6
7.5	8	10
8	7	8.8
8.5	6	7.5
9	2	2.5

### Descriptive statistics

The mean total score of the IPOS Neuro-S8 was 8.0 (SD = 4.8, range 0–23). Kurtosis (0.2) and skewness (0.7) suggested a near normal distribution, confirmed by visual inspection of the histogram. The highest-scoring item was *spasms* (Table 2). Visual inspection of item-specific histograms and skewness statistics revealed positive skewness (±0.5) for all items except *pain* and *spasms*, which exhibited normal distributions. Floor effects were evident for all items, with 20.0% to 97.5% of patients reporting the lowest possible score of 0. Ceiling effects were observed only for *spasms*, where 17.5% of patients achieved the highest possible score of 4.

**Table 2.** Descriptive statistics and item distribution (*n* = 80)

		*n* (%)				
	Mean (SD)	0	1	2	3	4
Pain (“Schmerzen”)	1.73 (1.34)	21 (26.3)	14 (17.5)	19 (23.8)	18 (22.5)	8 (10.0)
Shortness of breath (“Kurzatmigkeit”)	0.61 (0.91)	50 (62.5)	15 (18.8)	11 (13.8)	4 (5.0)	–
Nausea (“Übelkeit”)	0.36 (0.86)	66 (82.5)	4 (5.0)	5 (6.3)	5 (6.3)	–
Vomiting (“Erbrechen”)	0.04 (0.25)	78 (97.5)	1 (1.3)	1 (1.3)	–	–
Constipation (“Verstopfung”)	1.00 (1.30)	43 (53.8)	13 (16.3)	10 (12.5)	9 (11.3)	5 (6.3)
Mouth problems (“Symptome im Mund”)	0.72 (1.07)	48 (60.0)	15 (18.8)	10 (12.5)	5 (6.3)	2 (2.5)
Spasms (“Spastik”)	2.14 (1.35)	16 (20.0)	5 (6.3)	25 (31.3)	20 (25.0)	14 (17.5)
Difficulty in sleeping (“Schlafstörungen”)	1.25 (1.29)	32 (40.0)	17 (21.3)	15 (18.8)	11 (13.8)	5 (6.3)

### Validity

**Figure 1.**
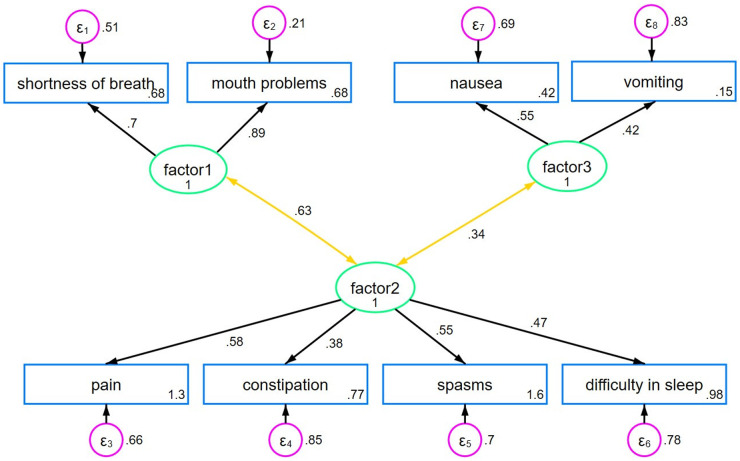
Three-factor CFA model.

Based on clinical reasoning and visual inspection of the scree plot, we selected a 3-factor-solution for the EFA. The first factor, *breath-mouth connection*, included 2 items with an eigenvalue of 2.50. The second factor, *pain-sleep cycle*, encompassed 4 factors and had an eigenvalue of 1.32. The third factor, *nausea-vomiting link*, consisted of 2 items with an eigenvalue of 0.98. These 3 factors explained 60.1% of the total variance. The suitability of the data for factor analysis was confirmed by the Bartlett’s test (*p* < 0.001) and the Kaiser-Meyer-Olkin Measure of Sampling Adequacy (KMO = 0.67). The results of the rotated loadings on each of the 3 factors are shown in Table 3 (the results of the DWLS analysis can be found in the Supplemental File S2 and are comparable, yet not identical). We applied a 3-factor-model to our data for the CFA, as shown in Figure 1 and Table 4. Factors 1 and 2 were moderately correlated with each other (*r* = 0.63; *p* < 0.001), while factor 2 and factor 3 were weakly correlated (*r* = 0.34; *p* = 0.128), suggesting associated but distinct constructs. All fit indices indicated a good model fit.

**Table 3.** Rotated factor loadings of the German version of the IPOS Neuro-S8

	Factor		
	1	2	3
Shortness of breath (“Kurzatmigkeit”)	0.99		
Mouth problems (“Symptome im Mund”)	0.57		
Difficulty in sleeping (“Schlafstörungen”)		0.57	
Spasms (“Spastik”)		0.52	
Pain (“Schmerzen”)		0.43	
Constipation (“Verstopfung”)		0.37	
Vomiting (“Erbrechen”)			0.86
Nausea (“Übelkeit”)			0.30

**Table 4.** Standardized factor loadings (standard error, SE), factor correlation (SE), and the fit indices in the confirmatory factor analysis of the German version of the IPOS Neuro-S8

	Factor 1 (2 items)		Factor 2 (4 items)		Factor 3 (2 items)	
	*Standardized factor loading [95% CI]*	*SE*	*Standardized factor loading [95% CI]*	*SE*	*Standardized factor loading [95% CI]*	*SE*
Shortness of breath	0.70	0.10				
	[0.50 to 0.91]					
Mouth problems	0.89	0.11				
	[0.67 to 1.11]					
Pain			0.58	0.12		
			[0.36 to 0.81]			
Constipation			0.44	0.13		
			[0.14 to 0.63]			
Spasms			0.55	0.12		
			[0.32 to 0.78]			
Difficulty in sleeping			0.47	0.12		
			[0.23 to 0.71]			
Nausea					0.55	0.32
					[−0.08 to 1.19]	
Vomiting					0.42	0.25
					[−0.08 to 0.91]	
Correlation	*r*(factor 1, factor 2) = 0.63					
	*r*(factor 2, factor 3) = 0.34					
Fit statistics	χ^2^(18) = 15.76; *p* = 0.610					
	RMSEA = 0.00 (95% CI [0.00; 0.09])					
	CFI = 1.00					
	SRMR = 0.07					

SE = Standard Error, CI=Confidence Interval, RMSEA = Root Mean Square Error of Approximation, CFI = Comparative Fit Index, SRMR = Standardized Root Mean Square Residual, χ^2^: Likelihood ratio test of model vs. saturated.

Correlation analyses revealed robust associations between the IPOS Neuro-S8 and HOPE+ (*r_s_*(78) = 0.71, *p* < 0.001) and moderate correlations with the HALEMS (*r_s_*(78) = 0.48, *p* < 0.001). As hypothesized, the IPOS Neuro-S8 displayed a stronger correlation with the physical domain of the HALEMS (*r_s_*(78) = 0.51, *p* < 0.001) compared to the nonphysical domain (*r_s_*(78) = 0.36, *p* < 0.001) as physical symptoms predominate in the IPOS Neuro-S8.

### Reliability

Cronbach’s alpha, assessing internal consistency, yielded a value of 0.67 (acceptable). The same value was reported by Gao et al. (2016).

The test–retest reliability analysis indicated moderate agreement between baseline and 3-month follow-up, with an ICC value of 0.75 (95% CI: 0.63–0.84).

### Sensitivity to change

**Figure 2.**
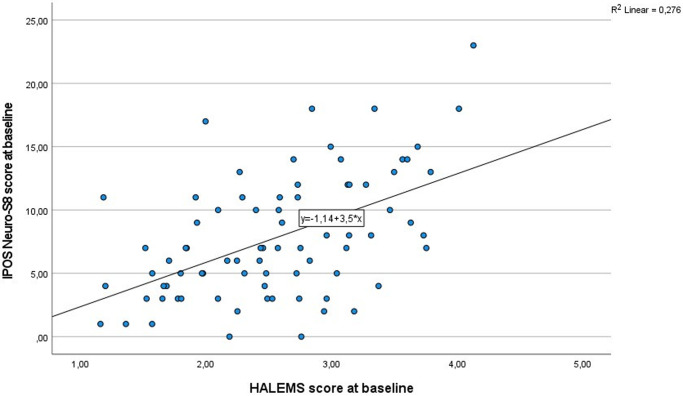
Linear regression for the IPOS Neuro-S8 and HALEMS.

The relationship between the IPOS Neuro-S8 and HALEMS led to the linear regression line equation of *y* = 1.14 + 3.5 × *x* and an *r*^2^ statistic of 0.28 (Figure 2). By calibrating the MCID for the HALEMS to 0.38, the MCID for the IPOS Neuro-S8 was determined to be 1.33 (=0.38 × 3.5).

## Discussion

The objective of the present study was to assess the reliability and validity of the IPOS Neuro-S8 in severely affected MS patients in the German healthcare setting. Our findings offer significant insights into the psychometric characteristics of the German IPOS Neuro-S8, highlighting its potential effectiveness in evaluating palliative care symptoms among severely affected MS patients as a prominent example of a neurological long-term condition.

The EFA revealed a structure comprising multidimensional symptom domains with 3 clearly defined and consistent factors. Notably, “shortness of breath” and “mouth problems” were grouped under *breath-mouth connection*, reflecting the causal connection between breathlessness and subsequent mouth-related issues such as dryness due to mouth breathing. Factor 2 included “constipation,” “spasms,” “pain,” and “difficulty in sleeping,” which clinically intertwine with constipation aggravating spasms, leading to pain and sleep disturbances (Cheatle et al. 2016; Coletti 2022), so were subsumed under *pain-sleep cycle*. In our study, we included severely affected individuals with MS who experience severe spasms, which can impact proper bowel function, leading to constipation (Wiesel et al. 2001). This explains why “spasms” and “constipation” were grouped as one factor, unlike the results of Gao et al. (2016), who did not exclusively focus on this patient group. “Vomiting” and “nausea” loaded onto the third factor, *nause-vomiting link*, indicating their close relationship, with nausea often preceding vomiting (van Rensburg 2008). Our study did not replicate the 2-factor structure reported by Gao et al. (2016), which may be due to a casemix/selection bias (chance). However, our findings suggest that factors 1 and 3 are distinct, aligning with clinical reasoning since the items grouped under each factor relate to 2 separate organic and functional systems (breathing vs. digestion). Conversely, the low to moderate intercorrelations between factors 2 and 3, as well as between factors 1 and 2, suggest distinct yet somewhat related dimensions, and all contribute to the overarching construct of general symptom burden in severe MS patients requiring palliative care.

Strong evidence supporting convergent validity was exemplified by a notable correlation with the HOPE+, which is revered as the current gold standard outcome measure within the German healthcare setting concerning palliative care assessment for neurological symptoms (Dillen et al. 2019). This correlation underscores the effectiveness of the IPOS Neuro-S8 in aligning with established standards, affirming its utility and relevance within the German healthcare context and enhancing confidence in its applicability for clinical and research purposes.

Furthermore, the IPOS Neuro-S8 and the HALEMS total score correlated only reasonably well. Given that the HALEMS encompasses physical and nonphysical dimensions, a strong correlation between these 2 measures was not initially expected, confirming discriminant validity, which was further supported by the low correlation between the nonphysical subscore of the HALEMS and IPOS Neuro-S8. As predicted, the association between the IPOS Neuro-S8 and the physical symptoms component of the HALEMS was notably stronger compared to its correlation with nonphysical aspects, although the former association fell short of the expected magnitude. Thus, while convergent validity could be supported by its association with the HOPE+, it could not fully be confirmed by the physical subscore of the HALEMS. The only moderate association observed between the IPOS Neuro-S8 and the physical domain of the HALEMS could be attributed to the fact that the HALEMS has not been specifically tailored to address the unique needs of individuals in need of palliative care or those severely impacted by their illnesses. Consequently, it might inherently encompass a different spectrum of physical needs than those addressed by the IPOS Neuro-S8, which is explicitly designed for palliative care contexts. Additionally, the sample selection in the current study could have influenced the ability to detect greater associations with the HALEMS as the IPOS Neuro-S8 was developed for a broad spectrum of neurological conditions. This study focused on a selective sample of severely affected MS patients, some of whom received MS-specific escalating immunotherapeutic agents while others no longer had MS-specific treatment options available besides symptomatic therapy (for subgroup details, see Golla et al. 2022). MS is often described as the chameleon of neurological diseases, as it can affect any part of the central nervous system, resulting in a wide range of symptoms that overlap with those seen in other inflammatory, vascular, and neurodegenerative neurological disorders. In this sense, MS can be considered a representative neurological disease. Further research including a broader spectrum of neurological conditions and refinement of assessment tools may be necessary to improve the convergent validity and underline its generalizability to other neurological populations.

The study revealed that the IPOS Neuro-S8 exhibited satisfactory internal consistency, as evidenced by a Cronbach’s alpha of 0.67, despite having only 8 items. This indicates that all items effectively evaluate symptom burden in palliative care patients with severe MS. The internal consistency observed underscores the reliability of capturing the diverse symptomatology experienced by this population. Notably, the internal consistency of the English version of the IPOS Neuro-S8, as reported by Gao et al. (2016), fell within a comparable range. These findings affirm the instrument’s cross-cultural applicability and value for clinical assessment and research within the MS palliative care context.

The test–retest reliability analysis yielded moderately correlated values, indicating consistency in measurements over time. The data were mean-centered to reduce the impact of treatment effects and clinical fluctuations, allowing for a more accurate assessment of the instrument’s reliability in capturing the symptoms and needs of severely affected MS patients. Gao et al. (2016) found reduced correlation coefficients in their broader sample during a 6-week follow-up period. Our findings indicate that the IPOS Neuro-S8 captures individual fluctuations in symptom burden over time, reliably captures individual fluctuations in symptom burden, and reliably gauges symptomatology and care needs, even with variations in treatment regimens or clinical states. The IPOS Neuro-S8 provides a nuanced evaluation of symptoms over time, aiding tailored interventions and optimizing patient care within the context of MS palliative care.

Last, we proposed a criterion for MCID for the IPOS Neuro-S8, which can be used to evaluate the sensitivity to change. This is crucial for longitudinal assessment in palliative care settings.

## Conclusion

The findings from this study shed light on the promising psychometric properties exhibited by the German version of the IPOS Neuro-S8 in evaluating palliative care concerns among severely affected MS patients. It provides clinicians with a reliable tool for assessing symptoms in MS patients with palliative care needs. Its consistent measurement of symptoms supports informed decision-making and tailored interventions, while solid correlations with established measures enhance its validity for clinical use. By elucidating the reliability and validity of this assessment tool within the context of MS palliative care, this research contributes significantly to the literature on effective evaluation instruments for neurological patients requiring palliative care.

## Strengths and limitations

In this study, we have strictly adhered to the recommendations and guidelines outlined in the POS manual (Antunes et al. 2012), which allows for comparison on an international level. It is, however, important to also recognize its limitations. While we met the minimum recommended sample size for a psychometric validation study of 10 subjects per item (Antunes et al. 2012), the resulting type II error may account for some of the nonsignificant statistical findings. Also, focusing on severely affected MS patients some of whom are receiving MS-specific treatment options (as this was a secondary analysis of a larger study), may limit the generalizability of its findings to other MS populations with milder disease severity levels or other neurological conditions. Additionally, the study’s confinement to the Cologne region may restrict broader applicability. Future research should replicate the study with more diverse neurological populations encompassing neurological conditions and disease severities to enhance generalizability and utility in diverse healthcare settings. Investigating the IPOS Neuro-S8 across different cultural and linguistic contexts is also needed, as cultural factors can influence symptom expression and care needs. Such an approach could provide valuable insights into its cross-cultural applicability and validity.

## Supporting information

Dillen et al. supplementary material 1 Dillen et al. supplementary material

Dillen et al. supplementary material 2 Dillen et al. supplementary material

## Supplementary material

The supplementary material for this article can be found at https://doi.org/10.1017/S1478951525000392.

## Notes

**KOKOS-MS trial group**

Anne Dorr, University of Cologne, Faculty of Medicine and University Hospital, Department of Palliative Medicine, Cologne, Germany

Beatrix Münzberg, University of Cologne, Faculty of Medicine and University Hospital, Department of Neurology, Cologne, Germany

Clemens Warnke, Philipps University Marburg and Department of Neurology, University Hospital Gießen and Marburg, Baldingerstraße, Marburg, Germany and University of Cologne, Faculty of Medicine and University Hospital, Department of Neurology, Cologne, Germany

Dirk Müller, University of Cologne, Faculty of Medicine and University Hospital, Institute for Health Economics and Clinical Epidemiology (IGKE), Cologne, Germany

Dorthe Hobus, University of Cologne, Faculty of Medicine and University Hospital, Department of Palliative Medicine, Cologne, Germany

Gundula Palmbach, University of Cologne, Faculty of Medicine and University Hospital, Clinical Trials Centre Cologne (CTCC), Cologne, Germany

Heidrun Golla, University Medical Center Göttingen, Department of Palliative Medicine, Göttingen, Germany and University of Cologne, Faculty of Medicine and University Hospital, Department of Palliative Medicine, Cologne, Germany

Isabel Franke, University of Cologne, Faculty of Medicine and University Hospital, Department of Palliative Medicine, Cologne, Germany

Kim Dillen, University of Cologne, Faculty of Medicine and University Hospital, Department of Palliative Medicine, Cologne, Germany

Martin Hellmich, University Medical Center Göttingen, Department of Medical Statistics, Göttingen, Germany and University of Cologne, Faculty of Medicine and University Hospital, Institute of Medical Statistics and Computational Biology (IMSB), Cologne, Germany

Monika Höveler, University of Cologne, Faculty of Medicine and University Hospital, Department of Neurology, Cologne, Germany

Solveig Ungeheuer, University of Cologne, Faculty of Medicine and University Hospital, Department of Palliative Medicine, Cologne, Germany

Sophia Kochs, University of Cologne, Faculty of Medicine and University Hospital, Department of Palliative Medicine, Cologne, Germany

Veronika Dunkl, University of Cologne, Faculty of Medicine and University Hospital, Department of Palliative Medicine, Cologne, Germany

Yasemin Göreci, University of Cologne, Faculty of Medicine and University Hospital, Department of Neurology, Cologne, Germany
