# Supplementary material for: Cross-cultural validation of the integrated palliative outcome scale for neurological patients (IPOS-Neuro S8) in multiple sclerosis patients
Source: Palliat Support Care. 2025 Jun 3;23:e110. doi: 10.1017/S1478951525000392 (PMC13166662; doi:10.1017/S1478951525000392)
Supplement: Dillen et al. supplementary material 1 — Dillen et al. supplementary material [file S1478951525000392sup001.docx]

**Supplemental Table 2.** Rotated factor loadings of the German version of the IPOS Neuro-S8 using Diagonally Weighted Least Squares (DWLS)

|  | Factor | | |
| --- | --- | --- | --- |
|  | 1 | 2 | 3 |
| Shortness of breath („Kurzatmigkeit“) | 0.78 |  |  |
| Mouth problems (“Symptome im Mund”) | 0.91 |  |  |
| Difficulty in sleeping („Schlafstörungen“) |  | 0.41 |  |
| Spasms („Spastik“) |  | 0.62 |  |
| Pain („Schmerzen“) |  | 0.63 |  |
| Constipation („Verstopfung“) |  | 0.48 |  |
| Vomiting („Erbrechen“) |  |  | 0.66 |
| Nausea („Übelkeit“) |  |  | 0.89 |
