# Supplementary material for: Cross-cultural validation of the integrated palliative outcome scale for neurological patients (IPOS-Neuro S8) in multiple sclerosis patients
Source: Palliat Support Care. 2025 Jun 3;23:e110. doi: 10.1017/S1478951525000392 (PMC13166662; doi:10.1017/S1478951525000392)
Supplement: Dillen et al. supplementary material 2 — Dillen et al. supplementary material [file S1478951525000392sup002.docx]

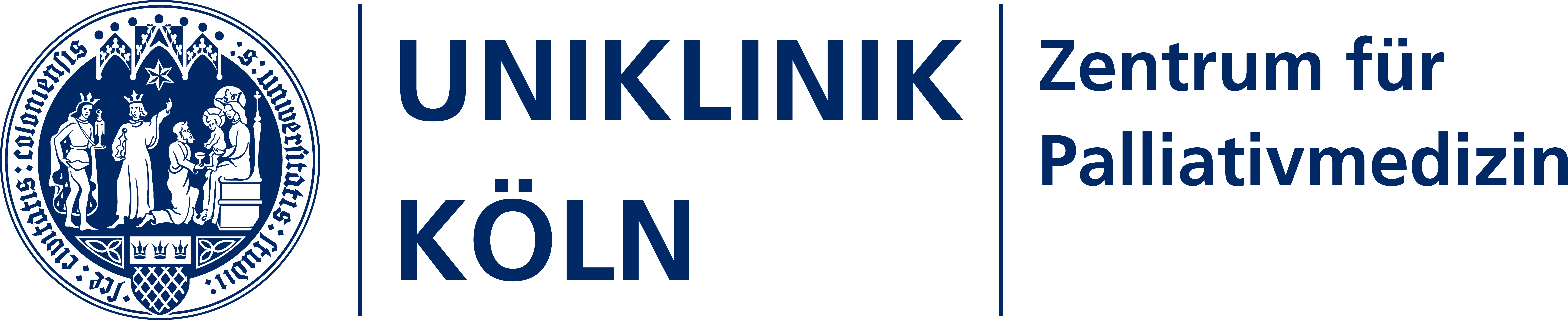

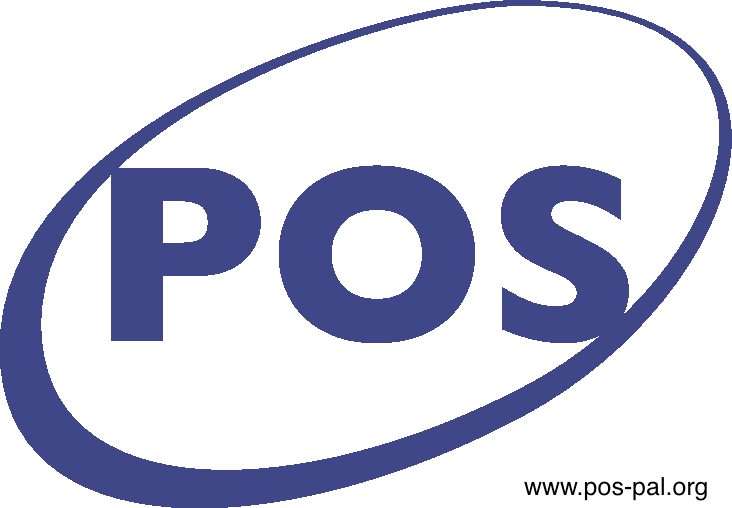

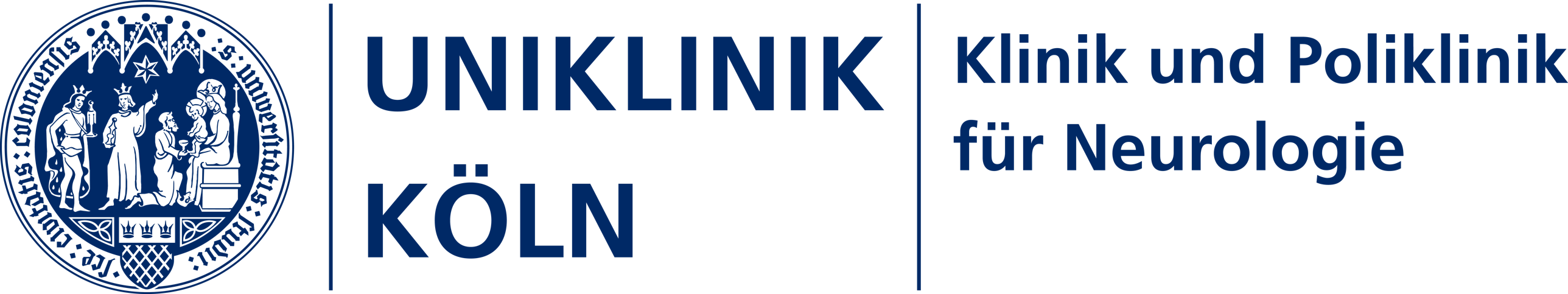
**IPOS Neuro-­‐S8 Patienten Version**

# F1. Welche Beschwerden oder Anliegen haben/hatten Sie in den letzten 3 Tagen?

1..........................................................................................................................................................

2..........................................................................................................................................................

3. ........................................................................................................................................................

# F2. Im weiteren Verlauf befindet Sie eine Liste an Symptomen, die Sie ggf. haben. Bitte kreuzen Sie für jedes Symptom jeweils ein Kästchen an und beurteilen Sie, wie stark Sie sich durch das Symptom in den letzten 3 Tagen beeinträchtigt gefühlt haben.

|  | **Gar nicht** | **Ein wenig** | **Mäßig** | **Schwer** | **Sehr schwer** |
| --- | --- | --- | --- | --- | --- |
| **Schmerzen** | 0□ | 1□ | 2□ | 3□ | 4□ |
| **Kurzatmigkeit** | 0□ | 1□ | 2□ | 3□ | 4□ |
| **Übelkeit (das Gefühl, erbrechen zu müssen)** | 0□ | 1□ | 2□ | 3□ | 4□ |
| **Erbrechen** | 0□ | 1□ | 2□ | 3□ | 4□ |
| **Verstopfung (Darmträgheit)** | 0□ | 1□ | 2□ | 3□ | 4□ |
| **Symptome im Mund (z.B. Mundtrockenheit, Apthen)** | 0□ | 1□ | 2□ | 3□ | 4□ |
| **Spastik (Muskelkrämpfe)** | 0□ | 1□ | 2□ | 3□ | 4□ |
| **Schlafstörungen** | 0□ | 1□ | 2□ | 3□ | 4□ |

|  | **Alleine** | **Mit Hilfe eines Freundes/einer Freundin oder eines**  **Angehörigen/einer Angehörigen** | **Mit Hilfe eines Mitarbeiters/einer Mitarbeiterin** |
| --- | --- | --- | --- |
| **F3. Wie haben Sie den Fragebogen ausgefüllt?** | 1□ | 2□ | 3□ |

*Wenn Sie sich über einen der in diesem Fragebogen angesprochenen Punkte Sorgen machen, sprechen Sie bitte mit Ihrem Arzt/Ihrer Ärztin oder Ihrem Pflegepersonal.*

IPOS NEURO S8 IPOSNEUROS8-­‐V1-­‐DE 10/02/2021
